# Hydrogen Storage on a New 2D Orthorhombic Boron Nitride Allotrope: Insights from Density Functional Theory

**DOI:** 10.3390/nano16120765

**Published:** 2026-06-17

**Authors:** Talha Zafer

**Affiliations:** 1 Department of Materials Science and Engineering, Faculty of Mechanical Engineering, Delft University of Technology, Mekelweg 2, 2628 CD Delft, The Netherlands; t.zafer@tudelft.nl; 2 Vocational School of Health Services, Sakarya University, 54050 Sakarya, Turkey

**Keywords:** hydrogen storage, 2D orthorhombic diboron dinitride, o-B_2_N_2_, Li-functionalization, density functional theory, ab initio molecular dynamics, clean energy

## Abstract

Hydrogen is a clean and renewable energy carrier, but its reversible storage near ambient conditions remains a major challenge. Here, density functional theory (DFT) combined with ab initio molecular dynamics (AIMD) is employed to assess the newly predicted 2D orthorhombic diboron dinitride (o-B_2_N_2_) monolayer, in pristine and Li-functionalized forms, as a hydrogen storage medium. On the pristine surface, H_2_ physisorbs with binding energies of −0.158 to −0.174 eV. Li atoms anchor strongly at the hexagonal hollow sites ($E_{\mathrm{bind}}$ from −0.979 to −1.321 eV, strongest at the B-rich H1 site), donate 0.65–0.84 |e| to the substrate, and render the semiconducting monolayer metallic. A positive cluster formation energy (+0.171 eV per Li pair) and a 5 ps AIMD simulation at 400 K confirm that the Li adatoms remain dispersed, without clustering. Each ${\mathrm{Li}}^{+}$ center polarizes and binds up to five H_2_ molecules, with average adsorption energies of −0.207 to −0.336 eV/H_2_, within the optimal window for room-temperature reversible storage. The 4Li@o-B_2_N_2_(20H_2_) system attains a theoretical gravimetric capacity of 15.12 wt% and a practical capacity of 10.99 wt% under realistic operating conditions (charging at 30 atm/25 °C; release at 3 atm/100 °C). These results establish Li-functionalized o-B_2_N_2_ as a promising hydrogen storage material that merits experimental exploration.

## 1. Introduction

Given hydrogen’s status as an environmentally friendly and renewable energy source, there is a growing interest in replacing fossil fuels with hydrogen. To enable its widespread use in various energy-related technologies, hydrogen storage materials need to exhibit reversible hydrogen storage with high gravimetric and volumetric densities under ambient thermodynamic conditions. Additionally, the adsorption energy of hydrogen should be around 0.2–0.4 eV per H_2_ molecule, as this value represents an optimal adsorption enthalpy at room temperature [1]. This ensures strong hydrogen affinity, allowing for the storage of a significant amount of hydrogen gas at the charging pressure (around 30 bar), while still facilitating the release of most of the hydrogen at the discharge pressure (approximately 1.5 bar). However, achieving high gravimetric densities in solid-state hydrogen storage materials that can operate under ambient conditions poses significant challenges, with the U.S. Department of Energy targeting system-level capacities of 5.5 wt% (2025) and an ultimate goal of 6.5 wt% [2].

In recent years, significant research effort has been devoted to 2D nanomaterials as promising candidates for next-generation hydrogen storage media, primarily due to their large surface-to-volume ratio and outstanding geometrical and electronic properties [3]. However, most of these systems have exhibited weak adsorption energies for hydrogen, limiting their practicality as hydrogen storage media. To address this challenge, investigations have focused on metal element-decorated 2D sheets, which have shown a substantial increase in H_2_ adsorption energy. Recent computational studies have demonstrated promising theoretical capacities: Li-decorated C_2_N achieving 13 wt% with 10 wt% practical capacity [4], Li-functionalized C_4_N reaching 8.0 wt% [5], Li-decorated BC_6_N demonstrating 8.7 wt% [6], and Li-functionalized 2D polyaramid achieving 10.62 wt% [7]. However, carbon-based materials and nanostructured graphite systems often exhibit high cohesive energies and relatively low binding energies, leading to Li clustering. Conversely, many reported 2D materials have demonstrated insufficient weight percentages for practical device applications under realistic pressure–temperature conditions.

Subsequently, numerous studies have consistently demonstrated the potential of h-BN material, primarily for physical hydrogen (H_2_) storage and, to a lesser degree, for chemical hydrogen storage. However, compared to materials like Metal–Organic Frameworks (MOFs) (for physisorption hydrogen storage) and complex hydrides (for chemisorption hydrogen storage), the development of h-BN as a hydrogen storage material has been relatively limited [8]. Nevertheless, recent reports and first-principles studies have unveiled new possibilities, with BN nanostructures achieving hydrogen storage capacities between 6.5 and 8.65 wt% [9], Li-decorated T-BN monolayers demonstrating an ultrahigh capacity of 12.31 wt%, and Mg-doped BN nanotubes showing capacities up to 9.65 wt% [10]. Recent work has further extended this paradigm to boron-based monolayers: Li-/Na-/K-/Ca-decorated boron monoxide reaches up to 11.75 wt% theoretical capacity [11], and boron-vacancy-induced porous boron nitride monolayers exhibit competitive performance for both Li-ion and H_2_ storage [12]. Among the alkali- and alkaline-earth metals investigated for 2D-material decoration, lithium is the most extensively used dopant for hydrogen storage applications, for three reasons: (i) Li has the lowest atomic mass (6.94 a.m.u.) of all metals, which maximizes the gravimetric storage capacity; (ii) Li readily donates its single 2 s valence electron to the substrate, producing a strongly polarised ${\mathrm{Li}}^{\delta +}$ cation that induces favorable charge-quadrupole and charge-induced-dipole interactions with H_2_ molecules—the so-called Kubas-like polarization mechanism that yields adsorption energies in the optimal 0.2–0.4 eV window for room-temperature reversible storage; and (iii) Li-decorated 2D systems have been shown to support multiple H_2_ molecules per metal center (up to five H_2_ per Li atom) thanks to the small ionic radius of ${\mathrm{Li}}^{+}$ and the dominantly electrostatic nature of the Li-H_2_ interaction. These considerations motivate our choice of Li as the functionalizing species for o-B_2_N_2_. Despite the existing research studies in this field, there is currently a lack of comprehensive and detailed studies specifically focusing on the practical application of different graphene-like BN allotropes for hydrogen storage under realistic operating conditions.

Consequently, it is crucial and opportune to investigate the potential utilization of these recently discovered 2D BN allotropes, with particular emphasis on a newly identified 2D polymorph named o-B_2_N_2_, which exhibits an orthorhombic crystal lattice with a rearrangement of B and N atoms. In a noteworthy study conducted by Demirci et al. (2020) [13], density functional theory (DFT) calculations were employed to introduce and evaluate the dynamic and mechanical stability of the o-B_2_N_2_ monolayer. Additionally, ab initio molecular dynamics simulations were conducted, confirming the structural integrity of o-B_2_N_2_ at temperatures as high as 1000 K for a timescale of 10 ps. Notably, this newly identified 2D BN allotrope demonstrates semiconductor properties, characterized by a narrow direct bandgap of 0.64 eV, thereby presenting promising opportunities for various energy-related applications. While o-B_2_N_2_ remains a hypothetical structure, a plausible synthesis route was proposed by Demirci et al. (2020) based on its structural building blocks: precursors containing pre-formed B-B and N-N bonds, such as diboron tetrachloride (B_2_Cl_4_) and hydrazine (N_2_H_4_), can in principle react to yield -B-B-N-N- chains rather than the alternating B-N pattern in conventional h-BN. Matrix-isolation studies further confirm that B-B-N-N species form in low-temperature noble-gas matrices [14,15]. Subsequent deposition on a suitable 2D substrate could in principle produce monolayer o-B_2_N_2_; the experimental realization of this route is an open challenge that motivates further computational and experimental exploration. In the present study, we employed DFT calculations combined with realistic pressure–temperature thermodynamic analysis to investigate whether o-B_2_N_2_ satisfies the criteria for an optimal hydrogen storage medium, considering its potential for high theoretical gravimetric capabilities.

## 2. Computational Details

All calculations were performed using density functional theory (DFT) as implemented in the Vienna Ab Initio Simulation Package (VASP) [16]. The exchange-correlation interactions were treated within the generalized gradient approximation (GGA) using the Perdew–Burke–Ernzerhof (PBE) functional [17], and the projector augmented wave (PAW) method was employed to describe the interaction between ionic cores and valence electrons [18]. All geometry optimizations and total-energy calculations include Grimme’s DFT-D3 dispersion correction with Becke–Johnson damping (DFT-D3(BJ), IVDW = 12 in VASP) [19,20], applied uniformly to the pristine substrate, the isolated Li-atom and H_2_-molecule references, and all Li-decorated and H_2_-adsorbed configurations. This consistent treatment of dispersion interactions ensures that the binding-energy and adsorption-energy formulae are evaluated at the same level of theory across all subsystems, as is essential for accurate physisorption energetics. A plane-wave energy cutoff of 600 eV was used throughout all calculations, with convergence criteria for electronic self-consistency and ionic relaxation set to $10^{-6}$ eV and $10^{-3}$ eV ${Å}^{-1}$, respectively. The Brillouin zone was sampled using a Monkhorst–Pack k-point grid of 8 × 16 × 1 on the primitive cell (scaled appropriately, 4 × 8 × 1, for the 2 × 2 supercells used in adsorption studies and AIMD) [21]. The bandgap of 0.647 eV reported in Section 3.1 is the PBE-GGA value, consistent with the 0.64 eV originally reported by Demirci et al. (2020) at the same level of theory [13]; Demirci et al. further reported a hybrid-functional (HSE06) [22] bandgap of 1.70 eV for pristine o-B_2_N_2_. In the present work, the PBE-GGA functional was used throughout, since full HSE06 calculations on the Li-decorated supercells are computationally prohibitive. A vacuum layer of 20 Å in the z-direction was introduced to eliminate spurious interactions between periodic images. Charge transfer analysis was performed using the Bader charge decomposition method [23]. Ab initio molecular dynamics (AIMD) simulations were carried out in the NVT ensemble using a Nosé–Hoover thermostat [24,25] with a time step of 2 fs at 300, 400, and 600 K to assess the thermal stability of the pristine and Li-decorated o-B_2_N_2_ surfaces.

The adsorption energy of H_2_ molecules and the binding energy of Li atoms were calculated as

(1) $$E_{\mathrm{ads}}\left(H_{2}\right)=\frac{E\left(\mathrm{substrate}+nH_{2}\right)-E\left(\mathrm{substrate}\right)-nE\left(H_{2}\right)}{n}$$

and

(2) $$E_{\mathrm{bind}}\left(\mathrm{Li}\right)=\frac{E(\mathrm{substrate}+n\mathrm{Li})-E(\mathrm{substrate})-nE(\mathrm{Li})}{n}$$

where *n* represents the number of adsorbates and $E(H_{2})$, E(Li) are the energies of an isolated H_2_ molecule and Li atom, respectively, computed in a 15 Å cubic vacuum box with ISPIN = 2 for the spin-polarized Li reference. Negative values indicate stable adsorption/binding. For practical hydrogen storage evaluation under realistic pressure–temperature conditions, the adsorption/desorption behavior was analyzed using grand canonical ensemble statistics, where the number of adsorbed H_2_ molecules was computed as

(3) $$N=N_{s}\left[\frac{\xi -1}{\xi }\right]$$

with $N_{s}$ representing the number of available adsorption sites and ξ being the single-site grand canonical partition function:

(4) $$\xi =1+\exp\left[-\frac{E_{b}-\mu }{k_{B}T}\right]$$

The chemical potential μ of H_2_ molecules at given temperature and pressure conditions was calculated as

(5) $$\mu_{H_{2}}\left(T,P\right)=\Delta H-T\Delta S+k_{B}T\ln\left(\frac{P}{P_{0}}\right)$$

where $k_{B}$ is the Boltzmann constant, $P_{0}$ is the standard atmospheric pressure (0.1 MPa), and ΔH and ΔS represent the changes in enthalpy and entropy, respectively.

The gravimetric hydrogen storage capacity is computed as

(6) $$\mathrm{wt}\%=\frac{m(H_{2})}{m\left(H_{2}\right)+m\left(\mathrm{substrate}\right)+m\left(\mathrm{Li}\right)}\times 100$$

where $m(H_{2})$ is the total mass of all adsorbed H_2_ molecules, m(substrate) is the mass of the o-B_2_N_2_ host atoms in the supercell, and m(Li) is the total mass of the Li adatoms. Both substrate and Li mass contributions are explicitly included in the denominator.

## 3. Results and Discussion

### 3.1. Structural and Electronic Properties

We retain the designation o-B_2_N_2_ introduced by Demirci et al. (2020) [13] and used consistently in the subsequent literature [26,27], in which ‘o-’ refers to the orthorhombic atomic-pair pattern (B-B and N-N pairs) characteristic of the structure rather than to a 3D Bravais lattice. The 2D primitive cell is rectangular, with lattice parameters a=4.57 Å, b=2.50 Å. The structural and electronic properties presented in this section have been obtained at the DFT-D3(BJ) level.

**Figure 1:**
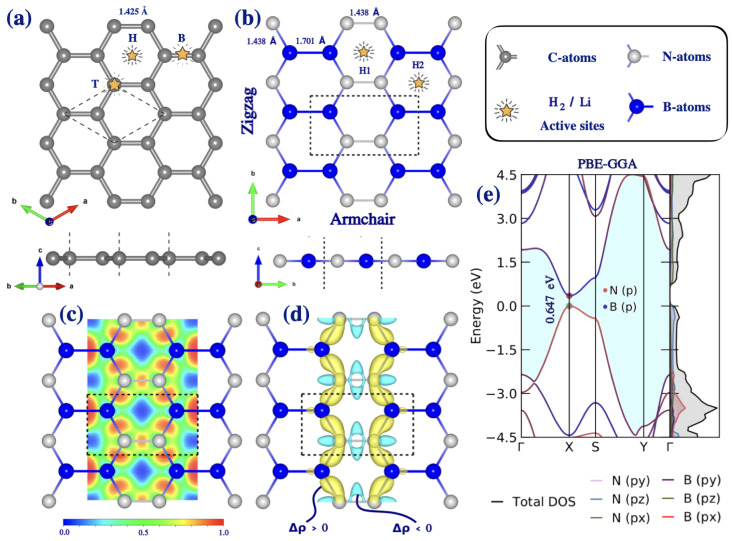
Structural and electronic properties of graphene and o-B_2_N_2_ monolayers. (**a**) Top and side views of graphene with high-symmetry adsorption sites labeled as hollow (H), top (T), and bridge (B), with C-C bond length of 1.425 Å. (**b**) Top and side views of o-B_2_N_2_ monolayer showing the rectangular primitive cell with two inequivalent hollow sites (H1 and H2), B-B and N-N bond lengths of 1.701 Å and 1.438 Å, respectively, and B-N cross-bonds of 1.438 Å, and zigzag/armchair directions indicated. (**c**) Electron localization function (ELF) map of o-B_2_N_2_, where the color scale (0.0–1.0) indicates low (blue) to high (red) electron localization. (**d**) Charge density difference of o-B_2_N_2_, with yellow (cyan) isosurfaces representing charge accumulation (Δρ>0) and depletion (Δρ<0), respectively. (**e**) Projected band structure and corresponding partial density of states (PDOS) of o-B_2_N_2_ calculated using PBE-GGA functional, showing a direct bandgap of 0.647 eV.

The structural analysis of the o-B_2_N_2_ monolayer reveals a distinctive orthorhombic lattice configuration that differs fundamentally from the conventional hexagonal boron nitride (h-BN) structure. As illustrated in Figure 1a,b, the o-B_2_N_2_ monolayer adopts a planar geometry with alternating B-N bonds arranged in a unique pattern that creates two distinct hexagonal ring types: B_4_N_2_ and B_2_N_4_ hexagons. This atomic arrangement results from the non-uniform distribution of boron and nitrogen atoms within the lattice, which fundamentally influences the electronic properties of the material.

The electron localization function (ELF) analysis presented in Figure 1c provides crucial insights into the bonding characteristics of the o-B_2_N_2_ monolayer. The ELF distribution reveals a pronounced asymmetry in electron localization between boron and nitrogen sites. The regions surrounding nitrogen atoms exhibit high ELF values (indicated by red zones), signifying strong electron localization, characteristic of the lone pair electrons on nitrogen. In contrast, the boron sites display lower ELF values (blue/green zones), reflecting the electron-deficient nature of boron atoms. This asymmetric electron distribution arises from the significant electronegativity difference between nitrogen (χ = 3.04) and boron (χ = 2.04) according to the Pauling scale [28,29]. The intermediate ELF values observed along the B-N bonds indicate the formation of polar covalent bonds with substantial ionic character, consistent with the mixed ionic–covalent bonding typical in boron nitride systems.

The charge density difference analysis depicted in Figure 1d further elucidates the charge redistribution upon bond formation. The yellow isosurfaces representing charge accumulation are predominantly localized around the nitrogen atoms and along the B-N bond axes, confirming significant electron transfer from boron to nitrogen. Conversely, the cyan regions indicating charge depletion are centered on the boron atoms, consistent with their role as electron donors. Using an isosurface value of 0.035 e/${Å}^{3}$, the calculated charge transfer reveals that each boron atom donates approximately 0.907 |e| to the neighboring nitrogen atoms, as confirmed by Bader charge analysis (Table 4). This substantial charge transfer creates local electric dipoles within the lattice that can serve as preferential binding sites for adsorbates, particularly polar molecules and alkali metal atoms.

The unique electronic structure of o-B_2_N_2_, characterized by its narrow direct bandgap of 0.647 eV, as shown in Figure 1e, distinguishes it from the wide-bandgap h-BN (experimentally ∼6 eV) and makes it particularly suitable for hydrogen storage applications. The projected density of states (PDOS) reveals that the valence band maximum is dominated by N-p orbitals, while the conduction band minimum has significant contributions from both B-p and N-p states. The presence of localized charge regions combined with the semiconducting nature of the material suggests favorable conditions for both physisorption and chemisorption processes, which are essential for reversible hydrogen storage under ambient conditions.

### 3.2. Binding Strength of H_2_-Molecules on Pure o-B_2_N_2_ Monolayer

**Figure 2:**
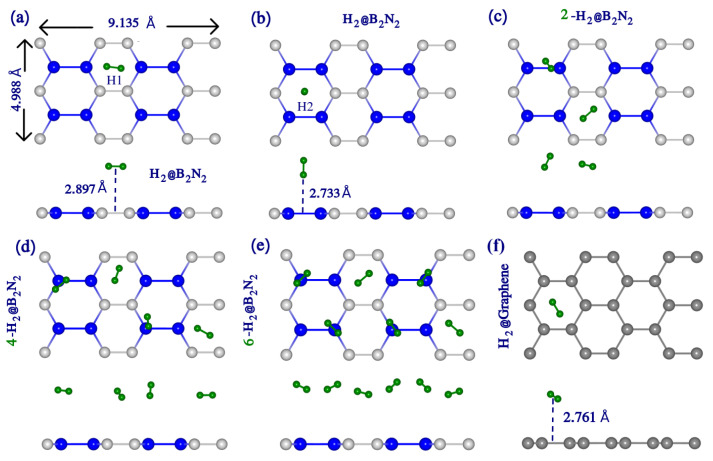
Optimized configurations of H_2_ molecules adsorbed on pristine o-B_2_N_2_ and graphene monolayers. Top and side views are shown for (**a**) single H_2_ at the H1 hollow site with binding height of 2.733 Å, (**b**) single H_2_ at the H2 hollow site with binding height of 2.897 Å, (**c**) 2-H_2_, (**d**) 4-H_2_, and (**e**) 6-H_2_ molecules adsorbed on o-B_2_N_2_. (**f**) H_2_ adsorption on graphene for comparison, with a binding height of 2.761 Å. The supercell dimensions are 9.135 Å × 4.988 Å. Green spheres represent H atoms.

As stated above, the new boron nitride allotrope o-B_2_N_2_ could be an optimal candidate for hydrogen storage owing to its outstanding properties—more particularly, its very high surface-to-volume ratio, very low molecular weight, and narrow electronic band gap compared to the hexagonal boron nitride, which exhibits an experimental band gap of approximately 6 eV. Therefore, we have first studied the interaction intensity between a H_2_ molecule and both o-B_2_N_2_ and graphene monolayers in their neutral state to clarify whether the neutral o-B_2_N_2_ monolayer can efficiently trap a H_2_ molecule or not. The optimized configurations for H_2_ adsorption on both substrates are presented in Figure 2.

The comparative analysis of H_2_ binding on pristine o-B_2_N_2_ and graphene monolayers, as summarized in Table 1, reveals several important distinctions that highlight the potential advantages of the o-B_2_N_2_ substrate for hydrogen storage applications. For single-H_2_-molecule adsorption on graphene, the binding energies vary depending on the adsorption site: the hollow (H) site exhibits the strongest binding at −0.168 eV, while both the top (T) and bridge (B) sites show weaker binding at −0.148 eV. These values fall within the typical physisorption range observed for carbon-based materials, where the weak van der Waals interactions dominate the adsorption mechanism.

In contrast, the o-B_2_N_2_ monolayer demonstrates more nuanced binding characteristics due to its heterogeneous surface topology. The presence of two distinct hexagonal ring types (B_4_N_2_ and B_2_N_4_) creates two inequivalent hollow sites designated as H1 and H2. The H2 site, centered on the B_2_N_4_ hexagon, exhibits a binding energy of −0.171 eV, which is comparable to the graphene hollow site within the typical numerical accuracy of DFT physisorption (∼0.01 eV). While the absolute binding magnitudes are similar, the heterogeneous surface topology of o-B_2_N_2_ provides two inequivalent hollow sites with distinct local electron-density environments—a feature absent on the homogeneous graphene surface—which could in principle influence adsorbate orientation and dipole/quadrupole alignment, and which motivates the Li-functionalization strategy explored in subsequent sections. The H1 site on the B_4_N_2_ hexagon shows a slightly weaker binding energy of −0.158 eV, consistent with the reduced electron density in the boron-rich region. We note that, in contrast to graphene, where the top (T) and bridge (B) sites are local minima distinct from the hollow (H) site, on o-B_2_N_2_, we found that initial configurations placing the H_2_ molecule directly above an atom (T-like) or above a B-N/B-B/N-N bond midpoint (B-like) relax during structural optimization toward the nearest H1 or H2 hexagonal-hollow configuration. This is a direct consequence of the corrugated charge-density landscape of the o-B_2_N_2_ surface, in which the alternating B-B and N-N pairs introduce sharp charge gradients along the bonds that disfavor atop and bridge physisorption, leaving the two hexagonal hollows as the only stable adsorption minima for molecular H_2_. The H1 and H2 entries in Table 1 therefore represent the complete set of physisorption minima on this surface.

The binding energy per H_2_ molecule remains essentially constant (within DFT numerical accuracy) as additional H_2_ molecules are adsorbed on the o-B_2_N_2_ surface, varying only by ∼4 meV between 2-H_2_ (−0.170 eV) and 6-H_2_ (−0.174 eV) configurations (Table 1). This invariance is itself a useful property: the maximum gravimetric capacity on the pristine surface (5.74 wt% with 6 H_2_) is achieved without any progressive weakening of the per-molecule binding, which would otherwise limit storage capacity. The intermolecular H_2_-H_2_ distances decrease from 3.128 Å (2-H_2_) to 2.694 Å (6-H_2_), suggesting increased packing density while maintaining molecular integrity, as evidenced by the constant H-H bond length of 0.751 Å across all configurations. The 6-H_2_ configuration represents a practical saturation of the pristine 2 × 2 supercell (9.135 × 4.988 ${Å}^{2}$): the average inter-H_2_ distance has already decreased to 2.69 Å, essentially at the van der Waals contact distance for H_2_ molecules (∼2.8 Å, twice the H_2_ kinetic radius). Accommodating a seventh H_2_ molecule within the same supercell would require either further compression below the van der Waals contact distance (introducing significant H_2_-H_2_ Pauli repulsion and a corresponding weakening of the per-molecule binding) or out-of-plane stacking, which is energetically unfavorable on a single-layer physisorbent, where each H_2_ molecule interacts only weakly with the surface. This physical limit on the pristine surface motivates the Li-functionalization strategy explored in subsequent sections, which introduces strong localized binding sites with adsorption energies in the optimal Kubas-like window and pushes the achievable capacity well beyond the pristine value.

The calculated desorption temperatures ($T_{D}$) provide crucial information regarding the practical applicability of these systems. For o-B_2_N_2_, the $T_{D}$ values range from 202 K to 223 K, which are marginally higher than those for graphene (189–215 K). While these temperatures remain below ambient conditions, they suggest that pristine o-B_2_N_2_ could maintain hydrogen adsorption under cryogenic conditions. The maximum gravimetric capacity of 5.742 wt% achieved with 6-H_2_ molecules on the pristine o-B_2_N_2_ surface is notable. However, the relatively low binding energies indicate that functionalization strategies are necessary to achieve reversible hydrogen storage under ambient conditions, motivating our investigation of Li-decorated systems in the following sections.

### 3.3. Binding Strength of Lithium on o-B_2_N_2_ Monolayer

**Figure 3:**
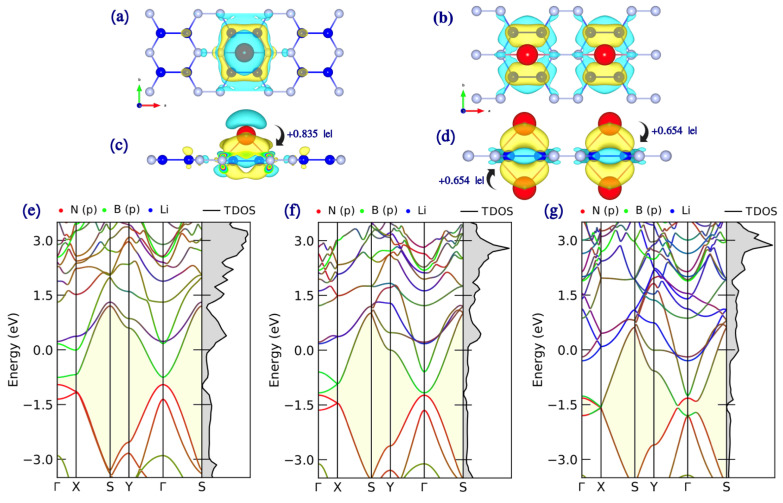
Lithium adsorption on o-B_2_N_2_ monolayer: structural and electronic properties. (**a**,**b**) Top views of single Li atom adsorbed on o-B_2_N_2_ with charge density difference isosurfaces (yellow: charge accumulation, cyan: charge depletion). (**c**) Side view showing charge transfer of +0.835 |e| from Li to the substrate. (**d**) Top and side views of 4Li@o-B_2_N_2_ configuration with charge transfer of +0.654 |e| per Li atom. Red spheres represent Li atoms. (**e**–**g**) Projected band structures with corresponding PDOS for 1Li@o-B_2_N_2_, 2Li@o-B_2_N_2_, and 4Li@o-B_2_N_2_ systems, respectively, demonstrating the semiconductor-to-metal transition upon Li functionalization.

The successful implementation of 2D materials for practical hydrogen storage applications critically depends on the stability of metal functionalization and the prevention of metal clustering, which would otherwise reduce the available surface area for H_2_ adsorption and compromise the storage capacity. In this section, we systematically investigate the binding characteristics of lithium atoms on the o-B_2_N_2_ monolayer to establish the thermodynamic stability of Li functionalization and assess the clustering propensity. The optimized structures and electronic properties of Li-adsorbed systems are presented in Figure 3.

The calculated binding energies presented in Table 2 demonstrate strong Li–substrate interactions that ensure stable functionalization. For single-Li-atom adsorption, the H1 site (centered on the B_4_N_2_ hexagon) exhibits a binding energy of −1.321 eV, stronger than the H2 site on the B_2_N_4_ hexagon (−0.979 eV). This site preference can be rationalized by the electronic structure of the o-B_2_N_2_ surface: the B_4_N_2_ hexagon, with its higher density of electron-deficient boron atoms, provides more favorable sites for accepting electron density from the Li atoms. The Li atom donates approximately 0.787 |e| to the substrate at the H1 site, as confirmed by Bader charge analysis, establishing a predominantly ionic interaction that anchors the Li atom to the surface.

A critical criterion for assessing clustering resistance is the comparison between Li–substrate binding energy and Li-Li cohesive energy. The cohesive energy of bulk lithium metal is approximately 1.63 eV/atom. The binding energies obtained for Li on o-B_2_N_2_ (−0.979 to −1.365 eV) are somewhat smaller in magnitude than this value, raising the legitimate question of clustering propensity that is addressed quantitatively below. The minimum Li-Li distances in our optimized structures range from 4.261 Å (4Li configuration) to 9.102 Å (1Li at H1), which are substantially larger than the Li-Li distance in bulk lithium (3.04 Å). This spatial separation, enforced by the geometric constraints of the preferred adsorption sites, is consistent with the absence of cluster nucleation, which we verify directly through cluster formation energy calculations and AIMD simulations below.

To address quantitatively the question of Li clustering on o-B_2_N_2_, we computed the cluster formation energy from two-Li configurations placed at maximum supercell separation (Li-Li =4.570 Å, “dispersed”) versus close proximity (initial Li-Li =2.800 Å, “clustered”, chosen to mimic incipient bulk-Li coordination):

(7) $$\Delta E_{\mathrm{cluster}}=E\left(2{\mathrm{Li}}_{\mathrm{clustered}}\right)-E\left(2{\mathrm{Li}}_{\mathrm{dispersed}}\right)=+0.171\;\mathrm{eV}.$$

The positive sign indicates that dispersion is thermodynamically preferred over clustering by 171 meV per Li pair. Two physical mechanisms underlie this preference. First, the substantial Li → o-B_2_N_2_ charge transfer (0.65–0.84 |e| per Li, Table 2) depletes the Li valence electrons, eliminating the metallic Li-Li bonding character that would otherwise drive cluster formation. Second, during the structural relaxation of the clustered configuration, one of the two Li atoms underwent a substantial in-plane displacement (8.3 Å, traversing the periodic boundary) before settling into a new minimum that was still 0.171 eV less stable than the dispersed reference—a clear signature of an active tendency to escape close Li-Li proximity. Thus, although $|E_{\mathrm{bind}}\left|\;<\;\right|E_{\mathrm{coh}}^{\mathrm{bulk}\;\mathrm{Li}}|$ in absolute terms, the explicit configurational energetics show that Li atoms favor dispersion on the o-B_2_N_2_ surface.

**Figure 4:**
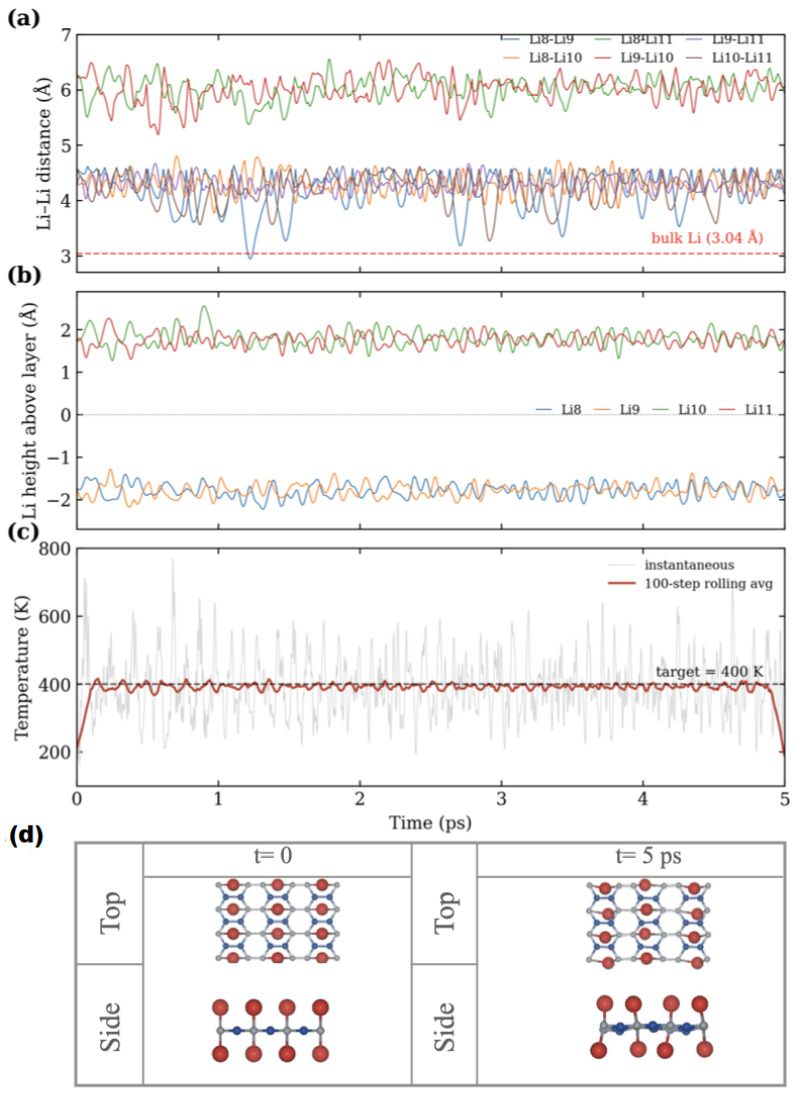
Ab initio molecular dynamics trajectory of the 4Li@o-B_2_N_2_ system at 400 K for 5 ps. (**a**) Li-Li distances as a function of time (six pairs); the bulk Li distance of 3.04 Å is shown as a horizontal red dashed line for reference. (**b**) Li atom heights relative to the substrate plane (Li10, Li11 above; Li8, Li9 below). (**c**) System temperature: instantaneous values (gray) and 100-step rolling average (red), with the target of 400 K indicated by a dashed line. (**d**) Top and side views of the initial (t=0, left) and final (t=5 ps, right) configurations of the trajectory, showing that all four Li atoms remain at their hexagonal-hollow adsorption sites on an intact, thermally fluctuating substrate. The simulation demonstrates that all four Li atoms remain bound to their adsorption sites and dispersed (mean Li-Li ≫ 3.04 Å) throughout the simulation, with the system temperature stabilizing rapidly around the target.

To further verify that the Li-decorated configuration is dynamically stable at hydrogen storage operating temperatures, we performed an AIMD simulation of the 4Li@o-B_2_N_2_ system at 400 K for 5 ps (NVT, Nosé–Hoover thermostat, 2 fs time step). The simulation reproduces the target temperature with mean 392.5 K (standard deviation 87.6 K), and the four Li atoms remain bound to their adsorption sites throughout the 5 ps. The mean Li-Li separations span 4.18–6.01 Å, well above the bulk Li distance of 3.04 Å (Figure 4). Although thermal fluctuations occasionally bring two adjacent Li atoms within 3.0 Å transiently, the system rapidly returns to the equilibrium dispersed configuration. No Li clustering, surface desorption, or substrate disruption is observed. These results, combined with the +0.171 eV cluster formation energy reported above, demonstrate that Li dispersion on o-B_2_N_2_ is both thermodynamically preferred and kinetically robust at operating temperatures. In particular, the AIMD trajectory provides a direct dynamical lower bound on the Li migration barrier: during the 5 ps simulation at 400 K, no Li atom is observed to leave its initial adsorption hollow or migrate to a neighboring site, indicating that the effective barrier for Li hopping is significantly larger than the thermal energy $k_{B}T\approx 34$ meV at this temperature. Combined with the positive cluster formation energy ($\Delta E_{\mathrm{cluster}}=+0.171$ eV per Li pair), which establishes a thermodynamic driving force against aggregation, and with the strong site-pinning effect of the Li-to-substrate charge transfer (+0.787 |e| per Li at H1, Table 2) that depletes the Li valence electron and eliminates Li-Li metallic bonding character, these three independent pieces of evidence—thermodynamic, dynamical, and electronic—together establish that the aggregation of Li adatoms on o-B_2_N_2_ is suppressed under hydrogen storage operating conditions. Moreover, the static potential-energy landscape itself provides a conservative estimate of the relevant energy scale for Li migration: any path taking a Li atom out of its H1 ground-state hollow must traverse regions of the surface at least as unfavorable as the neighbouring H2 hollow, which lies 0.342 eV higher in energy (Table 2); the effective hopping barrier out of H1 is therefore bounded from below by approximately 0.34 eV, an order of magnitude larger than $k_{B}T\approx 34$ meV at 400 K, fully consistent with the absence of any hopping event in the AIMD trajectory. We further note that, because the dispersed configuration is the thermodynamic ground state ($\Delta E_{\mathrm{cluster}}\;>\;0$), even hypothetical Li migration between equivalent H1 hollows would not lead to aggregation: there is no energetic driving force toward the clustered state for migration to feed. An explicit nudged-elastic-band (NEB) calculation of the H1↔H2 migration barrier is computationally intensive on the present 2 × 2 supercell and lies beyond the scope of the current study; it is, however, an interesting direction for future work, particularly in conjunction with finite-temperature free-energy sampling.

The binding energy per Li atom is essentially independent of coverage (−1.32 eV for 1Li to −1.37 eV for 4Li, within DFT numerical accuracy), indicating that Li atoms occupy independent surface sites without significant inter-Li interaction. This is consistent with the Li-Li separations of ≥4.2 Å enforced by the geometric arrangement of the preferred H1 hollow sites in the supercell. The substantial charge transfer from Li to the substrate (0.654–0.843 |e| depending on configuration) depletes the Li atoms of their valence electrons, thereby eliminating the metallic bonding character that would drive cluster formation.

The electronic structure modifications upon Li adsorption have profound implications for hydrogen storage. As indicated by the decreasing work function values from the pristine o-B_2_N_2_ to the Li-functionalized systems (Table 2), the surface becomes increasingly electron-rich. As shown in Figure 3e–g, all Li@o-B_2_N_2_ structures with different numbers of Li atoms exhibit metallic properties. This metallization arises from the donation of Li valence electrons to the substrate, which populates previously unoccupied conduction band states and enhances the polarizability of the surface, creating positively charged Li sites that can interact strongly with H_2_ molecules through electrostatic and charge-induced dipole mechanisms, as explored in the following section. We note that the semiconductor-to-metal transition reported here is at the PBE-GGA level. Full HSE06 band-structure calculations on the Li-decorated supercells are computationally prohibitive, but the metallization mechanism—Li → substrate charge transfer populating previously unoccupied conduction-band states—is expected to be qualitatively preserved at the hybrid-functional level.

### 3.4. Interaction of H_2_ with a Li-Functionalized o-B_2_N_2_

**Figure 5:**
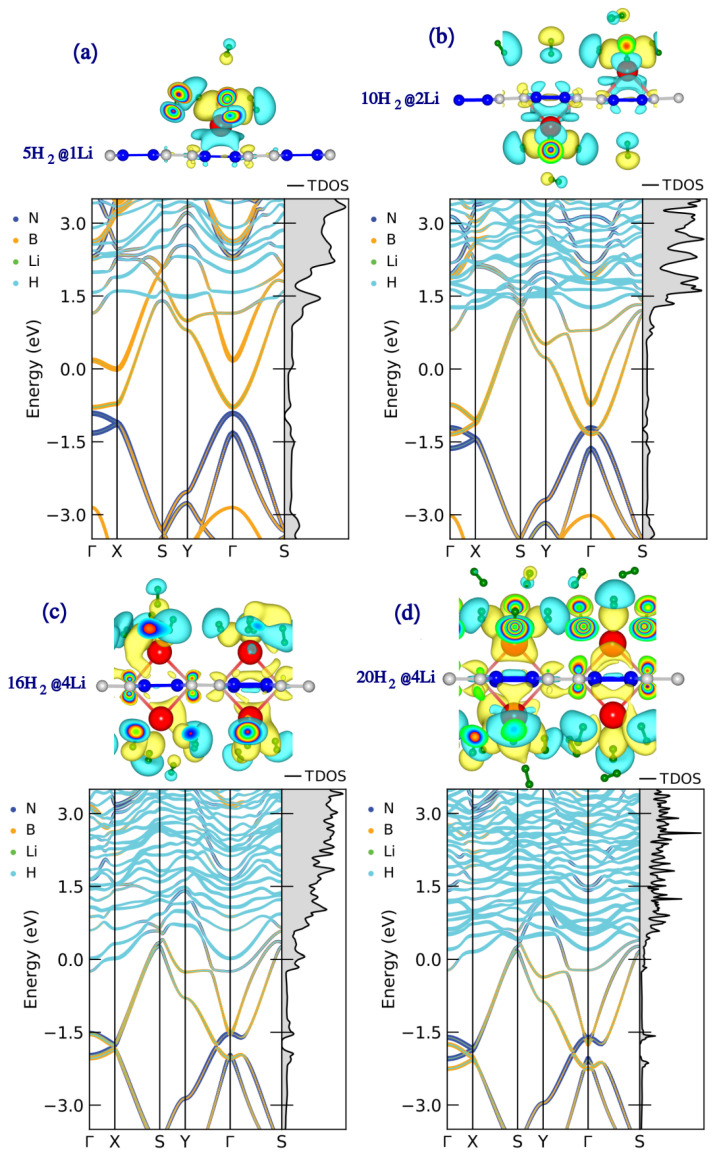
Hydrogen storage configurations on Li-functionalized o-B_2_N_2_ monolayer. Side views with charge density difference isosurfaces and corresponding projected band structures with PDOS for (**a**) 5H_2_@1Li-o-B_2_N_2_, (**b**) 10H_2_@2Li-o-B_2_N_2_, (**c**) 16H_2_@4Li-o-B_2_N_2_, and (**d**) 20H_2_@4Li-o-B_2_N_2_ representing the maximum theoretical capacity of 15.12 wt%. Yellow (cyan) isosurfaces indicate charge accumulation (depletion). Red spheres: Li atoms; green spheres: H atoms. The band structures show metallic character with multiple flat bands arising from H_2_ molecular states.

The Li-functionalization of o-B_2_N_2_ dramatically enhances its hydrogen storage capabilities, transforming it from a material with modest physisorption characteristics into a highly promising candidate for practical hydrogen storage applications. The optimized configurations for various Li-H_2_ systems along with their electronic structures are presented in Figure 5.

As presented in Table 3, the adsorption energies of H_2_ molecules on Li-functionalized o-B_2_N_2_ fall within the range of −0.207 to −0.336 eV, representing a substantial enhancement compared to the pristine surface (−0.158 to −0.174 eV). This range is particularly significant as it falls precisely within the thermodynamically optimal window for reversible hydrogen storage at ambient conditions [1].

The interaction mechanism between H_2_ molecules and Li-decorated o-B_2_N_2_ can be understood through the polarization model. Upon Li functionalization, the Li atoms become positively charged (approximately +0.65 to +0.84 |e|) due to charge transfer to the substrate, as established in Section 3.3. These cationic Li centers generate strong local electric fields that polarize approaching H_2_ molecules. The induced dipole moment in H_2_ interacts electrostatically with the ${\mathrm{Li}}^{+}$ center, resulting in binding energies that exceed those achievable through pure van der Waals interactions while remaining below the threshold for dissociative chemisorption. The preservation of the H-H bond length at 0.751 Å across all configurations (Table 3) confirms that the H_2_ molecules remain in their molecular form, which is essential for reversible storage.

The gravimetric hydrogen storage capacity exhibits a remarkable dependence on the Li loading and H_2_ coverage. Starting from the 1Li-H_2_ configuration with a modest capacity of 0.971 wt%, the system progressively achieves higher storage densities as more H_2_ molecules are accommodated around each Li center. Each Li atom can effectively coordinate with up to 5 H_2_ molecules in its immediate vicinity, resulting in a maximum capacity of 4.675 wt% for the 1Li-5H_2_ system. When both surfaces of the monolayer are functionalized with Li atoms (2Li and 4Li configurations), the storage capacity increases proportionally. The 2Li-10H_2_ system achieves 8.666 wt%, while the 4Li-20H_2_ configuration reaches an impressive theoretical gravimetric capacity of 15.12 wt%.

This maximum capacity of 15.12 wt% substantially exceeds many previously reported 2D material systems, including Li-decorated h-BN (6 wt%) [30], Li-decorated boron phosphide (7.402 wt%) [31], and Li-decorated borophene (6.80–11.49 wt%) [32,33]. The superior performance of Li@o-B_2_N_2_ can be attributed to the optimal combination of low substrate mass, high Li loading density enabled by the stable non-clustering behavior, and the ability of each Li center to coordinate multiple H_2_ molecules.

The microscopic origin of this advantage over graphene- and h-BN-based hosts deserves explicit comment. Both graphene and h-BN expose chemically homogeneous ring environments—uniform carbon hexagons in graphene and strictly alternating B-N hexagons in h-BN—on which a metal adatom interacts with a delocalised, weakly accepting π system; the resulting Li binding is weaker than the bulk Li cohesive energy by a wide margin, leaving such surfaces prone to metal aggregation and limiting the usable Li density. The B-B and N-N bond rearrangement of o-B_2_N_2_ breaks this homogeneity and creates two electronically inequivalent hollow sites, of which the electron-deficient, boron-rich B_4_N_2_ (H1) hexagon acts as a strong electron acceptor: Li binds there at −1.321 eV with a charge transfer of +0.787 |e| (Table 2 and Table 4), an essentially ionic anchoring that simultaneously suppresses clustering ($\Delta E_{\mathrm{cluster}}=+0.171$ eV) and generates an intense local electric field at the ${\mathrm{Li}}^{+}$ site. This field polarises approaching H_2_ molecules more effectively than the weaker Li centers on graphene- or h-BN-based hosts, placing the average adsorption energies (−0.207 to −0.336 eV/H_2_) squarely inside the optimal reversibility window, whereas Li@h-BN reaches only ∼−0.18 to −0.21 eV/H_2_ (Table 5). In addition, the narrow band gap of o-B_2_N_2_ (0.647 eV) allows the monolayer to become metallic upon Li adsorption, providing an efficient charge reservoir for the Li-to-substrate electron transfer, while the light B/N framework and the availability of H1 sites on both faces of the monolayer permit a dense, double-sided Li loading. The combination of stronger ionic Li anchoring, larger charge transfer, more effective H_2_ polarization within the optimal window, and low substrate mass is therefore the origin of the superior storage capacity of Li@o-B_2_N_2_ relative to graphene- and h-BN-based systems.

**Figure 6:**
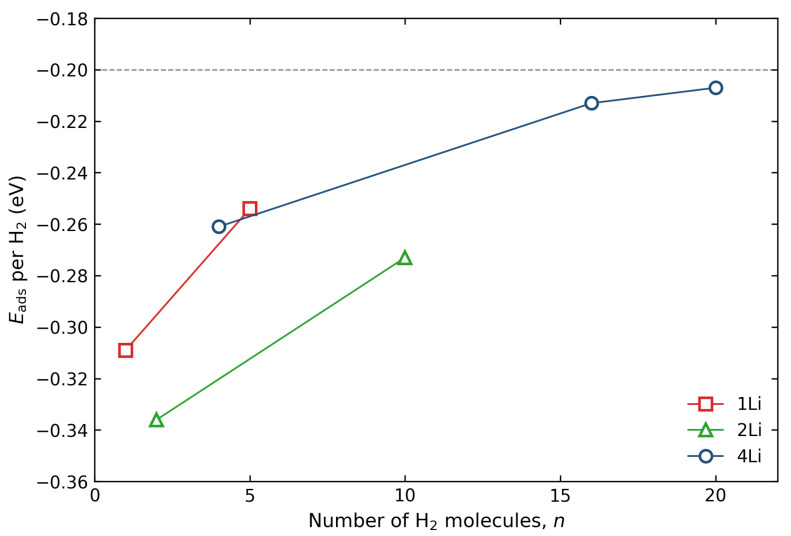
Average H_2_ adsorption energy per molecule, $E_{\mathrm{ads}}$, as a function of the total number of adsorbed H_2_ molecules (*n*) for 1Li, 2Li, and 4Li loadings on the o-B_2_N_2_ monolayer. The shaded green band indicates the optimal DOE window of −0.2 to −0.4 eV/H_2_ for room-temperature reversible storage. All configurations remain within this window; the H-H bond length is preserved at 0.751 Å throughout (Table 3), confirming that the H_2_ molecules remain in molecular form without dissociation.

The desorption temperatures calculated from the van’t Hoff equation range from 265 K (4Li-20H_2_) to 430 K (2Li-2H_2_), indicating that hydrogen release can be achieved under accessible temperature conditions. The dependence of the average H_2_ adsorption energy on coverage is summarized in Figure 6, which plots $E_{\mathrm{ads}}$ as a function of the total number of H_2_ molecules (*n*) for the three Li loadings considered (1Li, 2Li, and 4Li). All configurations lie within the optimal DOE window of −0.2 to −0.4 eV/H_2_, confirming that H_2_ remains in its molecular (physisorbed) form across the full coverage range; the constant H-H bond length of 0.751 Å reported in Table 3 provides direct structural evidence that no dissociation occurs even at the highest loadings. The decreasing trend in binding energy with increasing H_2_ loading (from −0.336 eV for 2Li-2H_2_ to −0.207 eV for 4Li-20H_2_) reflects the gradual saturation of the most favorable binding sites and the contribution of H_2_-H_2_ repulsive interactions at high coverage. This gradual weakening of binding at high loading is actually advantageous for practical applications, as it facilitates the release of stored hydrogen under moderate heating conditions while maintaining sufficient retention at ambient temperature and pressure.

**Figure 7:**
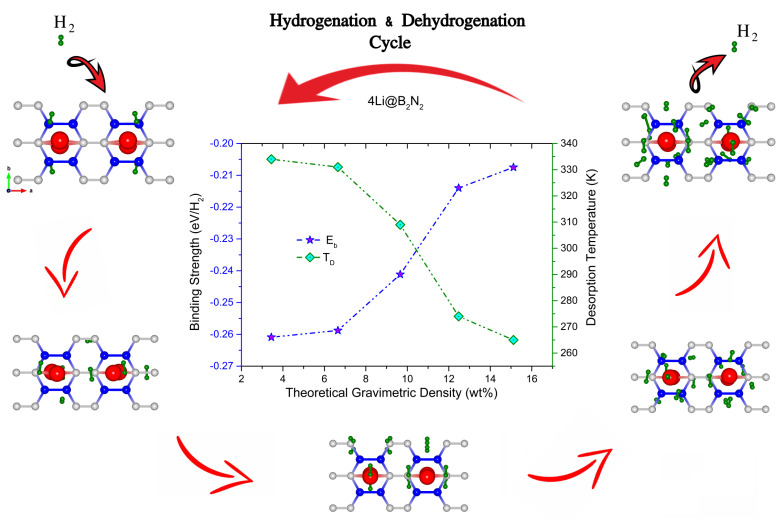
Hydrogenation and dehydrogenation cycle for the 4Li-o-B_2_N_2_ system. The central plot shows the average H_2_ binding strength ($E_{b}$, blue stars) and desorption temperature ($T_{D}$, green diamonds) as functions of theoretical gravimetric density. Surrounding snapshots illustrate the sequential adsorption of H_2_ molecules on the 4Li-functionalized o-B_2_N_2_ surface, demonstrating the reversible storage mechanism. Red spheres: Li atoms; green spheres: H atoms. The binding energy decreases from −0.26 eV to −0.20 eV as the gravimetric capacity increases from ∼3 wt% to ∼15 wt%, while desorption temperature correspondingly decreases from ∼340 K to ∼265 K.

The hydrogenation and dehydrogenation cycle for the 4Li-B_2_N_2_ system is illustrated in Figure 7, which clearly demonstrates the relationship between gravimetric capacity, binding energy, and desorption temperature.

### 3.5. Practical Operation Conditions of H_2_ Fuel Cell

While DFT calculations provide theoretical gravimetric H_2_ storage capacities, practical applications require a consideration of temperature (T) and pressure (P) effects. For physisorption-based H_2_ storage, where H_2_ gas molecularly adsorbs on the material surface through weak van der Waals forces, the binding probability follows equilibrium statistics as a function of both pressure and temperature. The adsorption/desorption behavior can be described using grand canonical ensemble statistics, with the partition function and chemical potential given by Equations (4) and (5), respectively, and the number of adsorbed H_2_ molecules computed using Equation (3).

**Figure 8:**
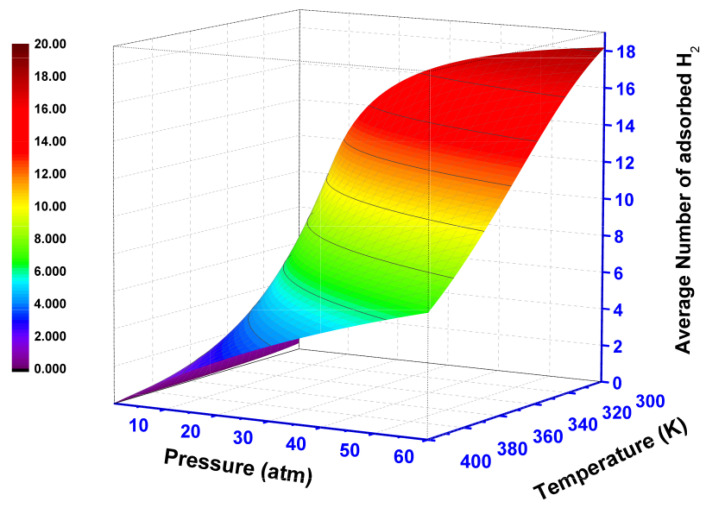
Three-dimensional N-P-T diagram showing the average number of adsorbed H_2_ molecules as a function of pressure and temperature for the 4Li-o-B_2_N_2_(20H_2_) system. The color gradient from blue to red indicates increasing H_2_ coverage from 0 to 20 molecules. High hydrogen uptake is achieved at low temperatures and high pressures, consistent with physisorption-dominated storage mechanism.

To quantitatively describe the H_2_ storage and release efficiency of the 4Li-B_2_N_2_ system, we have investigated and plotted the N-P-T diagram shown in Figure 8. For a viable hydrogen storage material, hydrogen molecules must be efficiently stored under charging conditions while being smoothly released under discharge conditions.

Considering the operational requirements of H_2_ fuel cells, the storage and release conditions are taken as 30 atm/25 °C and 3 atm/100 °C, respectively. Accordingly, the practical hydrogen storage capacity ($C_{\mathrm{prac}}$) is defined as the difference between the number of adsorbed hydrogen molecules under storage conditions ($N_{\mathrm{Ads}}^{H_{2}}$) and the number retained under release conditions ($N_{\mathrm{Des}}^{H_{2}}$).

**Figure 9:**
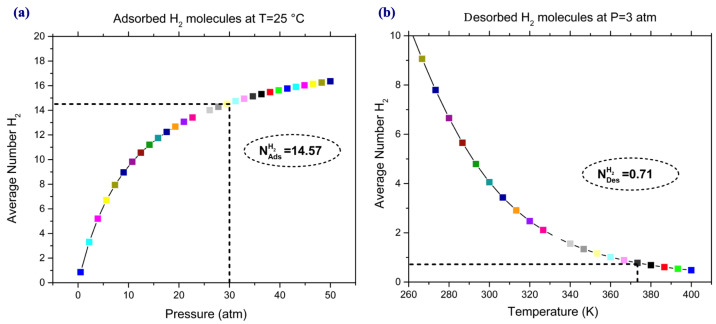
Hydrogen adsorption and desorption characteristics of 4Li-o-B_2_N_2_(20H_2_) system under practical operating conditions. (**a**) Number of adsorbed H_2_ molecules at room temperature (T = 25 °C) as a function of pressure. At the storage condition of 30 atm, $N_{Ads}^{H_{2}}$ = 14.57 molecules are stored. (**b**) Number of retained H_2_ molecules at release pressure (P = 3 atm) as a function of temperature. At 373 K (100 °C), only $N_{Des}^{H_{2}}$ = 0.71 molecules remain, indicating that 13.86 H_2_ molecules can be reversibly stored and released, corresponding to a practical gravimetric capacity of $C_{prac}$ = 10.99 wt%.

For the highest computed theoretical hydrogen storage configuration of the 4Li-B_2_N_2_ system (20 H_2_ molecules), it is clearly observed that the number of stored H_2_ molecules increases progressively with increasing external pressure at 25 °C (see the left panel of Figure 9). However, this increasing trend gradually slows down and nearly saturates at a pressure of 30 atm, yielding an adsorption capacity of $N_{\mathrm{Ads}}^{H_{2}}=14.57$.

In the case of hydrogen desorption at a fixed pressure of 3 atm, the number of retained H_2_ molecules decreases with increasing temperature (see the right panel of Figure 9). At 373.15 K (100 °C), the number of remaining hydrogen molecules is reduced to $N_{\mathrm{Des}}^{H_{2}}=0.71$. Consequently, 13.86 H_2_ molecules can be reversibly stored at 30 atm/25 °C and released at 3 atm/100 °C, corresponding to a practical storage capacity of $C_{\mathrm{prac}}=10.99$ wt%.

Our findings indicate that this practical capacity is reduced compared to the idealized DFT-predicted value (15.12 wt%). Nevertheless, it remains highly competitive when compared to other Li-functionalized two-dimensional lightweight materials, such as hexagonal boron nitride (h-BN, $C_{\mathrm{th}}=6$ wt%) [30], hexagonal boron phosphide (BP, $C_{\mathrm{th}}=7.40$ wt%) [31], borophene monolayers with theoretical capacities of 6.80 and 11.49 wt% [32,33], carbon nitride (CN, ∼10.81 wt%) [34], and dicarbon nitride (C_2_N, $C_{\mathrm{th}}=10$ wt%) [4]. Recent work on boron-based 2D materials further extends this comparison: Li-/Na-/K-/Ca-decorated boron monoxide reaches up to 11.75 wt% theoretical capacity [11], and boron-vacancy-induced porous BN monolayers exhibit competitive performance for both ion and H_2_ storage [12]. A summary comparison is presented in Table 5.

**Table 5:** Comparison of Li@o-B_2_N_2_ (this work) with recent state-of-the-art Li-functionalized 2D hydrogen storage materials.

Material	Theoretical *C* (wt%)	$E_{\mathrm{ads}}\left(H_{2}\right)$ Range (eV)	Reference
Li@h-BN	6.0	∼−0.18 to −0.21	[30]
Li@BP	7.40	∼−0.18 to −0.30	[31]
Li@borophene	6.80–11.49	∼−0.17 to −0.40	[32,33]
Li@C_2_N	13.0 (practical ∼10)	∼−0.21 to −0.28	[4]
Li/Na/K/Ca@BO	up to 11.75	−0.17 to −0.32	[11]
Li/Na/K@BN:VB (porous BN)	9.38–10.72	in optimal range	[12]
Li@o-B_2_N_2_ (this work)	15.12 (practical 10.99)	−0.207 to −0.336	—

Therefore, Li-functionalized 2D o-B_2_N_2_ monolayers are expected to be promising and highly efficient candidates for high-performance hydrogen storage applications.

## 4. Conclusions

In summary, we have systematically investigated the structural and electronic properties as well as the reversible adsorption and desorption mechanisms of hydrogen storage on a newly predicted 2D orthorhombic diboron dinitride (o-B_2_N_2_) monolayer using first-principles density functional theory calculations combined with ab initio molecular dynamics simulations. Our comprehensive analysis reveals that this novel boron nitride polymorph exhibits exceptional potential as a hydrogen storage medium, particularly when functionalized with lithium atoms.

The structural characterization of pristine o-B_2_N_2_ demonstrates a unique orthorhombic lattice with two distinct hexagonal ring types (B_4_N_2_ and B_2_N_4_), creating chemically heterogeneous binding sites. The electron localization function and charge density analyses reveal substantial ionic character in the B-N bonds, with a charge transfer of approximately 0.907 |e| from boron to nitrogen atoms, establishing localized charge regions that serve as preferential adsorption sites. The narrow direct bandgap of 0.647 eV distinguishes o-B_2_N_2_ from wide-bandgap h-BN and provides favorable electronic characteristics for adsorbate interactions.

For pristine o-B_2_N_2_, H_2_ molecules exhibit binding energies of −0.158 to −0.174 eV, which are comparable to or slightly higher than those on graphene (−0.148 to −0.168 eV). The maximum gravimetric capacity of 5.742 wt% achieved on the pristine surface demonstrates the intrinsic advantage of the low-mass BN framework, though the binding energies remain below the optimal range for ambient-temperature storage.

Lithium functionalization transforms the hydrogen storage characteristics dramatically. Li atoms bind at the H1 (B_4_N_2_, −1.321 eV) and H2 (B_2_N_4_, −0.979 eV) hexagonal hollow sites of o-B_2_N_2_, with the H1 site being preferred. Although the absolute binding magnitudes are smaller than the bulk Li cohesive energy (−1.63 eV), an explicit cluster formation-energy analysis ($\Delta E_{\mathrm{cluster}}=+0.171$ eV, dispersion preferred) and a 5 ps AIMD simulation at 400 K confirm that Li atoms remain dispersed and bound on the surface. The substantial Li-substrate charge transfer (0.65–0.84 |e|) eliminates the metallic Li-Li bonding character that would otherwise drive cluster formation, while creating cationic Li centers that strongly polarize and bind H_2_ molecules through electrostatic interactions while maintaining the molecular integrity of hydrogen.

The Li-functionalized o-B_2_N_2_ system achieves remarkable hydrogen storage performance. The average H_2_ adsorption energies of −0.207 to −0.336 eV fall precisely within the thermodynamically optimal range for reversible storage at ambient conditions. The maximum theoretical gravimetric capacity of 15.12 wt% (4Li-20H_2_ configuration) significantly surpasses previously reported 2D materials, including Li-decorated h-BN (6 wt%), boron phosphide (7.402 wt%), and borophene (6.80-11.49 wt%).

The practical hydrogen storage capacity, evaluated using grand canonical ensemble statistics under realistic operating conditions (storage: 30 atm/25 °C; release: 3 atm/100 °C) yields a reversible capacity of 10.99 wt%. This value substantially exceeds the material-only equivalents of the U.S. DOE targets (5.5 wt% for 2025 and 6.5 wt% ultimate, defined at system level), while remaining highly competitive among reported 2D hydrogen storage materials. The high gravimetric performance of Li@o-B_2_N_2_ demonstrates its viability as a candidate active material for practical fuel cell applications.

Our findings establish Li-functionalized o-B_2_N_2_ as an outstanding candidate material for high-capacity reversible hydrogen storage, combining the lightweight BN framework, stable Li functionalization without clustering, optimal H_2_ binding energetics, and exceptional gravimetric capacity. These results strongly motivate the experimental synthesis and characterization of this promising material system for next-generation clean energy storage technologies.

## Figures and Tables

**Table 1:** Binding energy ($E_{b}$), H-H bond length ($d_{H-H}$), binding height above the substrate ($h_{H_{2}-host}$), intermolecular H_2_-H_2_ distance ($d_{H_{2}-H_{2}}$), gravimetric capacity (wt%), and desorption temperature ($T_{D}$) of H_2_ molecules adsorbed on graphene and pristine o-B_2_N_2_ monolayers at the most favorable binding sites.

Systems	nH_2_	$E_{b}$	$d_{H-H}$	$h_{H_{2}-\mathrm{host}}$	$d_{H_{2}-H_{2}}$	wt	$T_{D}$
		(eV)	(Å)	(Å)	(Å)	(%)	(K)
Graphene	1-H_2_ (H)	−0.168	0.751	2.761	—	2.055	215
	1-H_2_ (T)	−0.148	0.751	2.785	—	2.055	189
	1-H_2_ (B)	−0.148	0.751	2.792	—	2.055	189
o-B_2_N_2_	1-H_2_ (H1)	−0.158	0.751	2.733	9.078	1.005	202
	1-H_2_ (H2)	−0.171	0.751	2.897	8.394	1.005	219
	2-H_2_	−0.170	0.751	2.818	3.128	1.990	217
	3-H_2_	−0.171	0.751	2.806	3.014	2.956	219
	4-H_2_	−0.172	0.751	2.797	2.875	3.902	220
	5-H_2_	−0.173	0.751	2.795	2.784	4.831	221
	6-H_2_	−0.174	0.751	2.784	2.694	5.742	223

**Table 2:** The binding energy of Li atoms ($E_{\mathrm{bind}}$, computed with DFT-D3(BJ)), the binding height of Li-atoms above the o-B_2_N_2_ surface, the minimum distance between two Li-atoms, charge transfer Q (|e|) and work function of lithium adsorbed on the most favorable binding site.

Systems	$E_{\mathrm{bind}}$	$d_{\mathrm{Li}-B_{2}N_{2}}$	$d_{\mathrm{Li}-\mathrm{Li}}$	$Q_{\mathrm{Li}}$	WF
	(eV)	(Å)	(Å)	(|e|)	(eV)
1Li@o-B_2_N_2_ (H1)	−1.321	1.671	9.102	+0.787	3.363
1Li@o-B_2_N_2_ (H2)	−0.979	1.704	9.081	+0.835	3.397
2Li@o-B_2_N_2_	−1.355	1.690	4.570	+0.843	1.508
4Li@o-B_2_N_2_	−1.365	1.732	4.261	+0.654	2.318

**Table 3:** The average binding strength per H_2_ molecule ($E_{b}$), H-H bond length $d_{H-H}$ (Å), binding height $d_{Li-H_{2}}$ (Å), charge transfer per H_2_ molecule $Q_{H_{2}}$ (|e|), gravimetric density wt (%), and desorption temperature $T_{D}$ (K) for the lowest/highest obtained storage capacities of the Li-functionalized o-B_2_N_2_ monolayer.

Hydrogenated	$E_{b}$	$d_{H-H}$	$d_{\mathrm{Li}-H_{2}}$	$Q_{H_{2}}$	wt	$T_{D}$
o-B_2_N_2_	(eV)	(Å)	(Å)	(|e|)	(%)	(K)
1Li-H_2_	−0.309	0.751	2.018	−0.032	0.971	395
1Li-5H_2_	−0.254	0.751	2.156	−0.024	4.675	325
2Li-2H_2_	−0.336	0.751	1.987	−0.035	1.862	430
2Li-10H_2_	−0.273	0.751	2.143	−0.026	8.666	349
4Li-4H_2_	−0.261	0.751	2.024	−0.031	3.440	334
4Li-16H_2_	−0.213	0.751	2.189	−0.022	12.473	273
4Li-20H_2_	−0.207	0.751	2.214	−0.019	15.119	265

**Table 4:** Bader charge analysis for o-B_2_N_2_ monolayer and its fully and partially lithiated states.

Systems	Average Charge State (|e|)			
	Li	B	N	H
o-B_2_N_2_	—	+0.907	−0.907	—
1Li@o-B_2_N_2_ (H1)	+0.787	+1.139	−1.225	—
1Li@o-B_2_N_2_ (H2)	+0.835	+1.133	−1.236	—
2Li@o-B_2_N_2_	+0.843	+1.054	−1.256	—
4Li@o-B_2_N_2_	+0.654	+1.006	−1.331	—
1Li@o-B_2_N_2_-5H_2_	+0.712	+1.098	−1.194	−0.024
2Li@o-B_2_N_2_-10H_2_	+0.768	+1.032	−1.218	−0.026
4Li@o-B_2_N_2_-20H_2_	+0.598	+0.987	−1.298	−0.019

## Data Availability

The original contributions presented in this study are included in the article; the numerical results of all DFT and AIMD calculations (binding energies, adsorption energies, Bader charges, gravimetric capacities, and the 4Li@o-B_2_N_2_ AIMD trajectory at 400 K) are reported in the figures and tables within the main text. The underlying VASP input/output files (POSCAR, INCAR, KPOINTS, OUTCAR, and AIMD trajectory files) are archived on the corresponding author’s institutional server and are available from the corresponding author upon reasonable request. Further inquiries can be directed to the corresponding author.

## Abbreviations

The following abbreviations are used in this manuscript:

DFT	Density Functional Theory
AIMD	Ab Initio Molecular Dynamics
GGA	Generalized Gradient Approximation
PBE	Perdew–Burke–Ernzerhof
PAW	Projector Augmented Wave
VASP	Vienna Ab Initio Simulation Package
HSE06	Heyd–Scuseria–Ernzerhof Hybrid Functional
DOE	U.S. Department of Energy
